# Fractal evolution under *in situ* pressure and sorption conditions for coal and shale

**DOI:** 10.1038/s41598-017-09324-9

**Published:** 2017-08-21

**Authors:** Rui Zhang, Shimin Liu, Yang Wang

**Affiliations:** 1 Department of Energy and Mineral Engineering, G3 Center and Energy Institute, The Pennsylvania State University, University Park, PA 16802 USA; 2 Key Laboratory of Coalbed Methane Resources and Reservoir Formation Process, Ministry of Education, Xuzhou, Jiangsu 221008 China; 3 School of Resources and Earth Science, China University of Mining and Technology, Xuzhou, Jiangsu 221116 China

## Abstract

Coalbed methane (CBM) and shale gas become two most important unconventional natural gas resources in US. The fractal dimension, known as the degree of self-similarity or irregularity, is an important parameter to quantitatively characterize gas storage capacity and gas transport properties in pores of rock matrix. In this study, two coal and two shale samples were evaluated to estimate fractal dimensions using combined small angle X-ray scattering (SAXS), small angle neutron scattering (SANS) and low-pressure N_2_ adsorption techniques. The results show that surface fractal dimension *D*_*s*_ of inaccessible pores is greater than that for total pores based on SANS results for all four tested samples. *D*_*s*_ of accessible pores estimated by N_2_ desorption is greater than that for N_2_ adsorption for each linear section of each tested sample. Based on *in situ* SANS results, *D*_*s*_ slightly decreases with increasing argon injecting pressure for San Juan coal. *D*_*s*_ decreases with increasing methane and CO_2_ injecting pressure for samples with high *D*_*s*_. However, *D*_*s*_ significantly increases when CO_2_ became liquid phase for samples with low *D*_*s*_. Furthermore, *D*_*s*_ almost didn’t change after methane and argon penetrations for all these samples except Marcellus outcrop shale.

## Introduction

Recent years, unconventional natural gas becomes one of the major energy resources since it is a carbon-less energy compared to the primary substitution of conventional fossil fuels such as coal and crude oil^1^. Among all the types of unconventional natural gas resources, the coalbed methane (CBM) and shale gas are two major gas resources which are known for large reserves and economically feasible for commercial production^2–5^. Both CBM and gas shale are self-sourced reservoir rocks, where the gas stores as free gas in both pores and fractures as well as the adsorbed gas in the nano-pores^4^. Both coal and shale are known to be fractal porous media which show either surface (boundary) fractal or mass (volume) fractal characteristics^6, 7^. The fractal dimension, also called the Hausdorff dimension, known as the degree of self-similarity or irregularity becomes an important parameter to characterize gas storage potential as well as gas transport properties^6–9^.

In the literatures, many techniques have been successfully used to characterize fractal dimension of rocks, such as scanning/transmission electron microscope (SEM/TEM)^10^, atomic force microscope (AFM)^11^, mercury intrusion porosimetry (MIP)^12^, low-pressure N_2_ adsorption^6, 13^, nuclear magnetic resonance (NMR)^14, 15^, ultra-/small angle X-ray scattering (USAXS/SAXS)^16–18^, and ultra-/small angle neutron scattering (USANS/SANS)^17, 19^. It was found that the fractal dimension of coal varied with gas pressure for different ranks based on SAXS data^20^. This is inconsistent with the pressurized lignite coal^21^ and helium-pressurization for bituminous coal^22^, where there is no discernable change of fractal dimension. The fractal dimension reduced with decreasing of particle size for anthracite using MIP^12^. It was interesting that the fractal dimension increased with increasing of particle size using AFM^11^ while decreased with increasing of particle size using SEM^10^ for the same bituminous coal. It was found the fractal dimension increased with increasing coal rank^20, 23^, where the fractal dimension estimated using MIP is greater than that for low-pressure N_2_ data^23^. Under heat treatment, the fractal dimension showed an initial increase with increasing of temperature and then followed with a decrease with continuous temperature increasing^20, 24^, finally increased with increasing tempearature^24, 25^. The extraction of organic micromolecules reduced the fractal dimension for coal^26^. Capillary condensation of water reduced the fractal dimension of pores in standstone^27^, which corresponded to the dehydration increased the fractal dimension of small pores in shale^16^. While this disagreed with lignite coal where the fractal dimension decreased after the dewatering^28^. Meanwhile, the weathering effect reduced the fractal dimension for shale^29^ and the fractal dimension changed after supercritical CO_2_-H_2_O treatment for coals^30^.

Recently, many researchers emphasized on the correlation analyses between the fractal dimension and rock properties as well as pore structure for shale^7, 13, 31–41^, which follow the methodology of a pioneer study using coal samples^6^. The results from shale rocks showed that the fractal dimension varied with both total organic carbon (TOC) and mineral compositions. Higher values of fractal dimension correlated with higher pore volume and surface area while lower average pore size for shale samples^7, 32, 35, 36, 38, 39^, which are consistent with the original coal study^6^. And it was found that the fractal dimension mainly corresponded with micropores^31^. In addition, the higher fractal dimension tends to have higher sorption capacity^7, 13^ and lower permeability^7^ for shale. While there is a U-shape between fractal dimension and sorption capacity for coal^6, 42^.

Based on previous mentioned literatures, extensive experimental studies of fractal characteristic have been carried out for both coal and shale. However, very few investigation evaluated the fractal features under *in situ* pressurized gas environment and the gas adsorption effect were also overlooked, especially for shale. As natural rocks, the pore can be either accessible or inaccessible to penetration fluids^43^. To our best knowledge, none effort has been reported to distinguish the fractal features of accessible and inaccessible pores under *in situ* gas pressurization and sorption environment. It is expected that the evolution of pore-rock interface and its irregularity with both gas pressure and adsorption effects is crucial for gas sorption behavior^13, 44^, diffusion^45^ and permeability^46^ evaluations for coal and shale.

In this study, we characterized the fractal dimensions for both accessible and inaccessible pores by using different lab techniques. The studied samples include San Juan sub-bituminous coal, Hazleton anthracite, Marcellus drilled core and outcrop shale samples. Uniquely, we dynamically evaluated the pressure dependent fractal dimensions under continuous hydrostatic gas injections along with gas adsorption influence. One goal of this study is to compare the fractal dimension estimated by different characterizations. The combined SAXS, SANS and low-pressure N_2_ sorption techniques were used to characterize the fractal features of the tested coals and shales. Another goal is to evaluate and compare the fractal dimensions of total, accessible and inaccessible pores. SANS and SAXS were used to determine the fractal dimension of total pores within the capable pore size range. Low-pressure N_2_ adsorption was used to evaluate the fractal dimension of accessible meso-/macro-pores, while the N_2_ desorption isotherm was used to evaluate the fractal dimension of accessible meso-/macro-pores with sorption hysteresis (dynamic-inaccessible) effect. SANS was used to evaluate the fractal dimension of inaccessible pores. Finally, the impact of gas pressurization and sorption effects on fractal dimensions were characterized and quantified. SANS was used to evaluate the evolution of fractal dimension of total pores during *in situ* argon pressurization and methane/CO_2_ adsorption floodings. In this study, the pressure dependent rock-fractal dimension evolution will add the knowledge of both gas storage and transport in coal and shale reservoirs.

## Results

### SAXS/SANS scatterings

**Figure 1:**
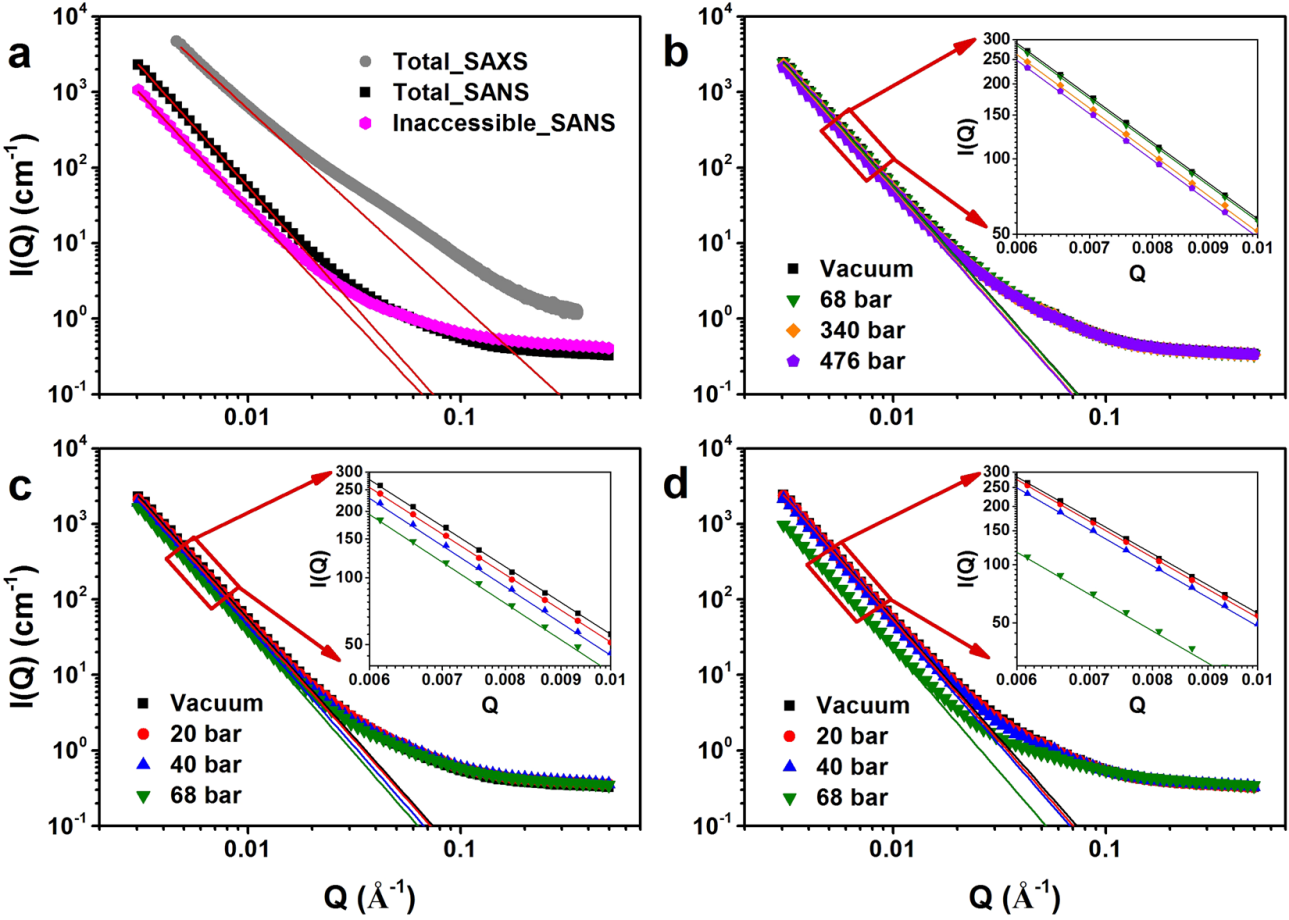
Typical scattering intensities (San Juan coal). (**a**) Scattering intensities of total pores detected by SAXS and SANS, scattering intensities of inaccessible pores detected by SANS; (**b**) scattering intensities during Ar penetration; (**c**) scattering intensities during CD_4_ penetration; (**d**) scattering intensities during CO_2_ penetration. (Note: The solid lines are modeled power law scattering intensities for fractal dimension determination.).

Figure 1 shows the scattering intensities *I*(*Q*) of San Juan coal containing SAXS and SANS results for total and inaccessible pores, SANS results for argon pressurization, and SANS results for methane and CO_2_ adsorption. It was found that all *I*(*Q*) decreased with increasing scattering vector *Q* for all tested conditions. *I*(*Q*) of SAXS is higher than that for SANS shown in Fig. 1a, which could be attributed to detectable inorganic matter-pore system for SAXS which generates more scattering intensity compared to SANS^17, 47, 48^. *I*(*Q*) of total pores is higher than that for inaccessible pores in the low *Q* range for SANS (Fig. 1a), suggesting that there is a certain percentage of accessible pores for San Juan coal^43^. For SANS measurements, *I*(*Q*) decreased with increasing injection pressure in the low *Q* range for argon, methane and CO_2_ (Fig. 1b–d). This intensity decrease for argon injection is primarily attributed to the pore shrinkage due to the solid skeleton contraction and could be combined with the pressure-induced mechanical compression on the solid skeleton that results in an increase of solid density due to the grain contraction (a schematic shown in Fig. 7 in Zhang *et al*.^43^). In addition to the mechanical compression effect as argon does, there is a sorption-induced effect on the microstructure of rocks for methane and CO_2_. As shown in Fig. 1c and d, the *I*(*Q*) decreases for methane and CO_2_ injections more severe than argon injection does. This is believed that there is a sorption induced gas-solid interface densification at which the sorption layers have higher density compared to the bulk gas, where this sorption-induced interface densification results in a reduction of the scattering contrast between pore and matrix^49, 50^. Interestingly, a huge decrease of scattering intensity for CO_2_ at 68 bar was observed as shown in Fig. 1d. One possible reason is CO_2_ became liquid phase at 68 bar at room temperature, where the density of liquid CO_2_ is significant greater than that of gaseous CO_2_. Thus, the scattering contrast between pore and rock matrix had a greater decrease compared to gaseous CO_2_. All the findings are shown similar for other three samples (Hazleton coal, Marcellus drilled core and outcrop shales) where scattering intensities were drawn in Figs S1, S2 and S3 in the supplementary. The only difference is scattering intensities of Hazleton coal shows a very small change varing by gas pressure even when CO_2_ became liquid (Fig. S1b–d). This could be caused by the extreme tight and complex of pore structure in anthracite, having the greatest TOC content of 91.14% (Table S1), compared with low rank coal or shale for adsorption^50^.

### Low-pressure N_2_ adsorption

**Figure 2:**
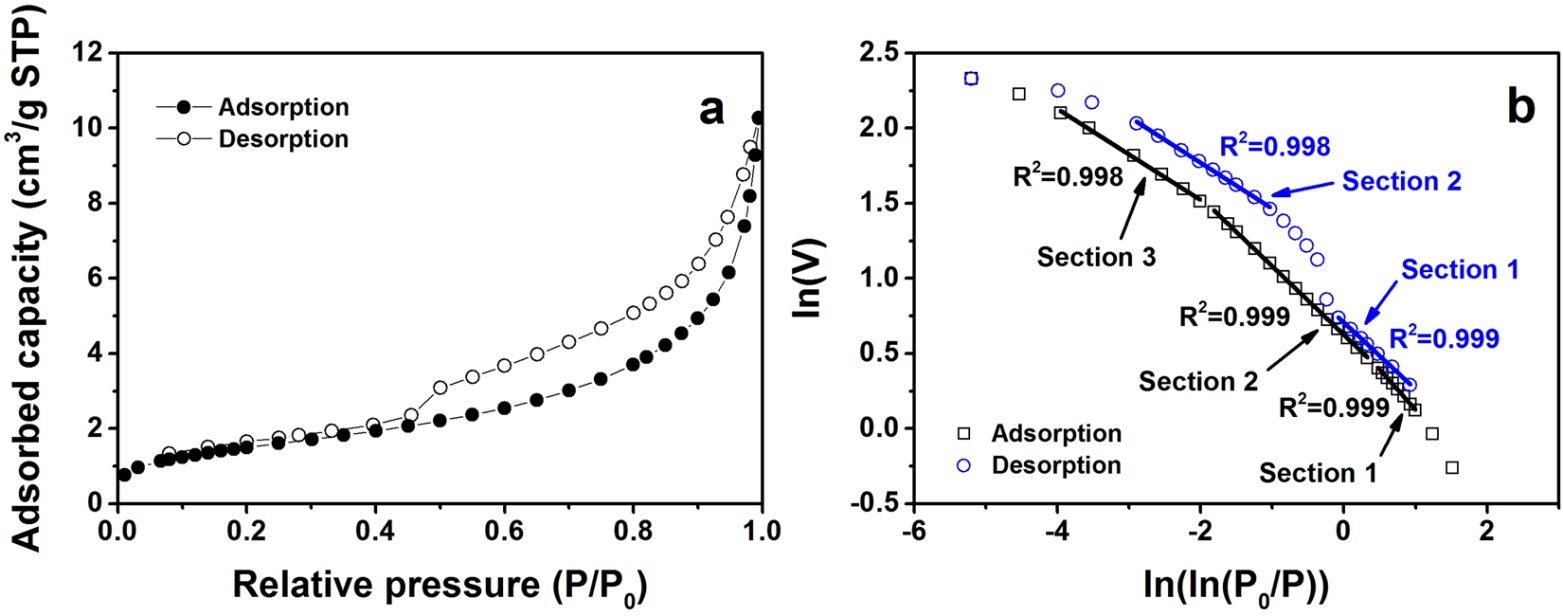
Typical low-pressure N_2_ sorption (San Juan coal). (**a**) Low-pressure N_2_ adsorption-desorption isotherms; (**b**) ln*V*-ln[ln(*P*_0_/*P*)] plot of N_2_ isotherms for fractal determination.

Figure 2 shows the low-pressure N_2_ adsorption-desorption isotherms and their ln(*V*)-ln(ln(*P*_0_/*P*)) plot for San Juan coal. It is noted that the hysteresis loop between adsorption and desorption isotherms could be the signature for the dominant effect of capillary condensation (Fig. 2a)^51^. The hysteresis of adsorption and desorption shows the type H3 loop^52^, which represents slit-shape pores for San Juan coal. All the findings are shown similar for other two samples (Marcellus drilled core and outcrop shales) where low-pressure N_2_ results were drawn in Figs S4 and S5 in the supplementary. The only difference is that Marcellus drilled core shale has a relative large hysteresis, while the Marcellus outcrop shale sample has a relative small one. This could be caused by different mineral compositions in drilled core shale compared with the weathered outcrop one. Marcellus drilled core sample has more complex pore structure with much smaller Quartz and more Muscovite contents (Table S1) compared with outcrop sample. Additionally, we failed to run Hazleton coal tested at The Pennsyvania State University and China University of Mining and Technology (Xuzhou) after a few trial measurements. Thus, we did not present the results of Hazleton anthracite here.

### Fractal characterization for total, accessible and inaccessible pores

**Figure 3:**
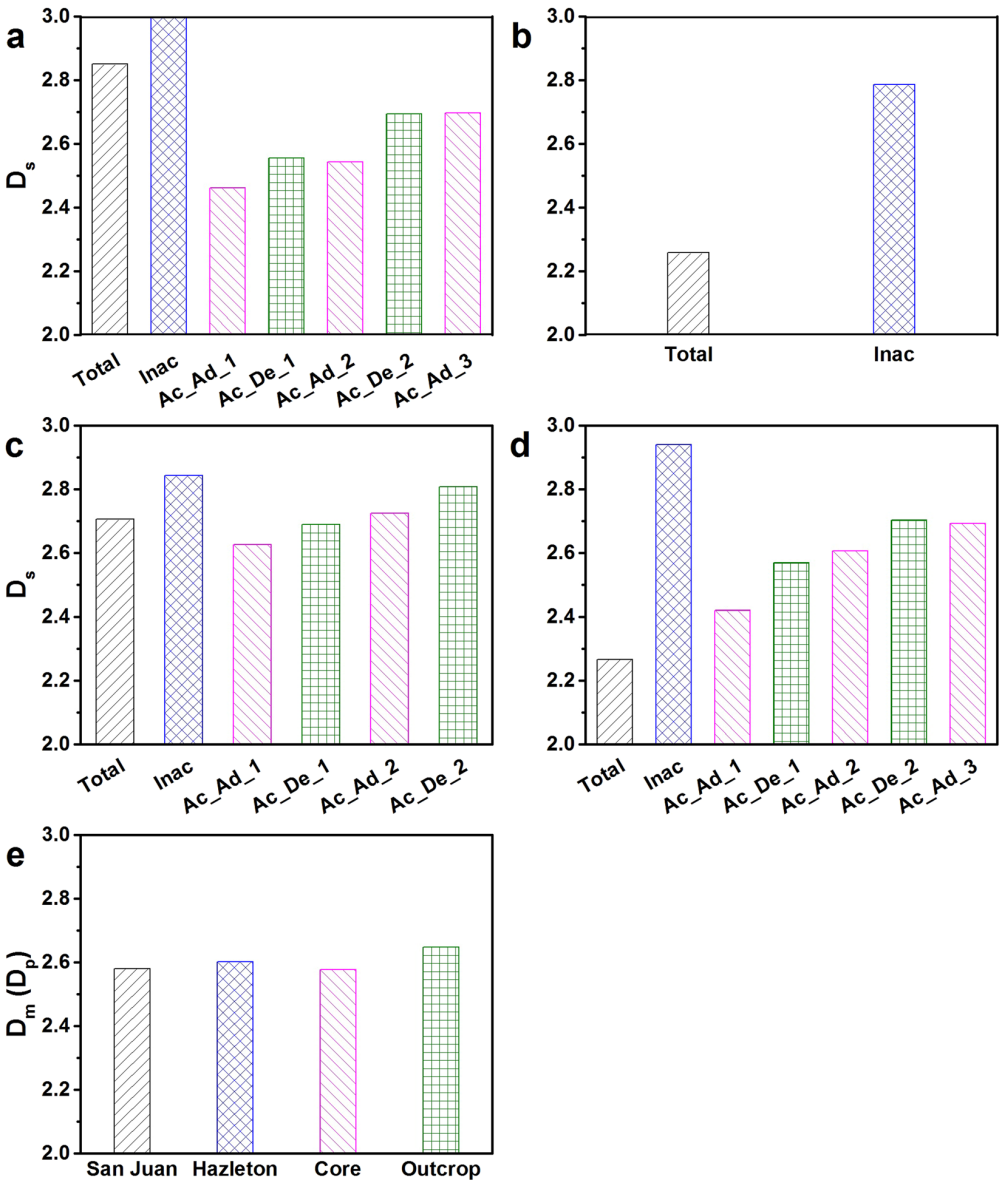
Fractal dimension from different techniques for total, accessible and inaccessible pores. (**a**) San Juan coal; (**b**), Hazleton coal; (**c**) Marcellus drilled core shale; (**d**) Marcellus outcrop shale; (**e**) vacuum condition of four samples using SAXS. (Note: Total: Total pores; Inac: Inaccessible pores; Ac_Ad_1: Accessible pores for linear section 1 of adsorption; Ac_De_1: Accessible pores for linear section 1 of desorption; Ac_Ad_2: Accessible pores for linear section 2 of adsorption; Ac_De_2: Accessible pores for linear section 2 of desorption; Ac_Ad_3: Accessible pores for linear section 3 of adsorption).

The scattering intensity *I*(*Q*) of inaccessible pores, detected at 340 or 476 bar using CD_4_ for SANS, shows smaller value compared to that for total pores at low *Q* region for each sample (Figs 1a, S1 a, S2 a and S3a). It is noted that highly pressurized CD_4_ at 340 or 476 bar was used to reach the zero average contrast (ZAC) condition for these tested rocks. The ZAC concept was used to quantify the pore accessibility for porous media, which has been successfully used for coal and shale in previous studies^9, 43, 49, 53–56^. The scattering at ZAC condition is contributed by pores which are inaccessible to CD_4_ based on two phase (pore-matrix) assumption^53^. According to this definition, *D*_s_ estimated at ZAC pressure (340 or 476 bar) is the surface fractal dimension from inaccessible pores which shows a higher value than that from total pores based on modeled results for each rock sample (Fig. 3a–d). The higher value of *D*_s_ for inaccessible pores is attributed to the higher heterogeneity of pore surface or irregularity of pore shape, which makes the pores tend to be disconnected with each other and thus they are inaccessible to penetrating fluids^43^. Here we want to point out that the physical property of inaccessible pore can influence the sorption hysteresis because the accessibility of the pore is pressure-dependent as we found in our previous studies^43, 44^.

It was found that the fractal dimension estimated by SAXS is a mass fractal (or pore fractal) rather than surface fractal compared to SANS results for all these samples, where the value of *D*_m_ (or *D*_p_) ranges between 2.58 and 2.65 (Fig. 3e). One reason is that the electron density detected by SAXS are different between organic and inorganic matters, while the scattering length density (SLD) detected by SANS are comparable for both organic and inorganic contents^48^. The mass fractal detected by SAXS combined both organic and inorganic matter-pore systems, while the surface fractal detected by SANS only represented organic pore system.

The *D*_s_ estimated by all linear sections of low-pressure N_2_ adsorption and desorption isotherms are smaller than that for total pores estimated by SANS for San Juan coal (Fig. 3a), since accessible pores are believed having smaller *D*_s_ compared with that for total pores and the *D*_s_ of inaccessible pores should be the greatest one. This is due to the pores with high roughness tends to be inaccessible for gases to penetrate into. However, for two shale samples, only *D*_s_ estimated by linear section 1 of N_2_ adsorption is smaller than that of total pores for Marcellus drilled core shale (Fig. 3c), and *D*_s_ estimated by all linear sections of both N_2_ adsorption and desorption are greater than that for total pores for Marcellus outcrop shale (Fig. 3d). This discrepancy could be caused by different probing length, different probing fluids (CD_4_ for SANS and liquid N_2_ for low-pressure adsorption), different detecting mechanisms for pore detection between low-temperature sorption and scattering techniques, as well as different chemical compositions between coal and shale rocks. The pore size range detected by N_2_ sorption is wider than that for small angle scattering experiments. This could give variations of comparison of *D*_s_ among total, accessible and inaccessible pores over the entire pore scale in rock matrix. In general, the scattering technique is a combined physical and chemical-based method, where the scattering occurred at the boundary (pore surface) between pore and solid matrix with different density and chemical composition. In contrast, the low-pressure sorption is a physical-based one, where the N_2_ sorption detects the geometry of the interface between the liquid film and vapor gas in pores which replicates the physical geometry of pore surface. It is important to note that (1) N_2_ has smaller molecular size compared to methane, where N_2_ molecules can penetrate into pores with smaller size which have relative greater surface fractal dimension. (2) Both Van der Waals and capillary force effects are coincident for the N_2_ adsorption experiment. The surface fractal dimension *D*_s_ may have a inequality relationship as 3(1 + *s*) ≤ *D*_s_ ≤ 3 + *s* ^57^, which suggests that *D*_s_ may be overestimated based on purely capillary condensation effect using correlation *s* = *D*_s_ − 3 based on Eq. 2 for low-pressure adsorption data analysis. Thus, the different detecting mechanisms may give variations for the comparisom of *D*_s_. In addition, San Juna coal has much higher TOC content (>70%) compared with two shale samples with TOC less than 10%. In this situation, the hypothesis: “*D*_s_ of accessbile pores <*D*_s_ of total pores <*D*_s_ of inaccessible pores” could not be valid for rocks with low carbon (high mineral) content such as shale in this study. Because the pore volume, pore shape and pore tortuosity could be different between organic carbon and mineral matters, where the essence of fractal dimension could be different. As a concenquence, further study should be considered.

For the low-pressure N_2_ sorption data, *D*_s_ estimated by desorption isotherm is greater than that for adsorption isotherm for each linear section for each sample (Fig. 3a–c). This *D*_s_ difference between adsorption and desorption could be interpreted as the hysteresis effect of N_2_ sorption. It is notable that all the sorption sites in accessible pores in the rock matrix are available during the adsorption process, while only several percentage of sorption sites are available for desorption process compared to adsorption occurrence^44^. This different availability of sorption sites between adsorption and desorption causes the relative higher desorption capacity compared to adsorption capacity at a certain pressure, which is usually called sorption hysteresis^44^. Sorptive fluids become hard to desorb and penetrate out of these pores with higher *D*_s_. Because pores with higher *D*_s_ potentially have more sorption sites and relative higher energy barrier, where fluids can be easily adsorbed into but hard to desorb and diffuse out of these highly fractal pores. Pores with higher *D*_*s*_ potentially have smaller accessibility/interconnectivity for penetrating fluids. It was pointed out that the estimated *D*_*s*_ might depend on experimental injecting pressure, sample type and instrument specifications. Further study focusing on the effects of fractal dimension on gas diffusion of coal and shale should be conducted to quantitatively evaluate the relationships between the estimated fractal dimensions and the gas transport properties.

In previous studies, researchers interpreted that the fractal dimension estimated by the N_2_ adsorption isotherm with relative pressure range between 0 and 0.5 represents the surface fractal dimension *D*_s_, while the pore fractal dimension *D*_p_ was estimated by the adsorption profile with relative pressure range between 0.5 and 1 for both coal and shale^6, 13, 31, 32, 36, 38, 40^. Based on this definition, the fractal dimension estimated by linear region 2 and 3 may be *D*_p_ rather than *D*_s_ for tested samples. In this situation, *D*_p_ estimated by desorption isotherm is greater than that for adsorption isotherm. It indicates that pores with higher *D*_p_ are potentially more irregular and may also have relative higher energy barrier, where fluids will be hard to diffuse out of these highly fractal pores. However, the *D*_p_ estimated by low-pressure N_2_ sorption is inconsistent with the results from SAXS based on tested three samples (San Juan coal, Marcellus drilled core and outcrop shales) in this study. Thus, the estimating methodology for surface and pore fractals are the same based on Frenkel-Halsey-Hill (FHH) model^58^ except different region of relative pressure, which may not be the case for estimation of different type of fractal dimensions. Further study need to consider the evidence for estimating both surface and pore fractals for porous rocks.

### Fractal evolution under *in situ* pressure and sorption effects

**Figure 4:**
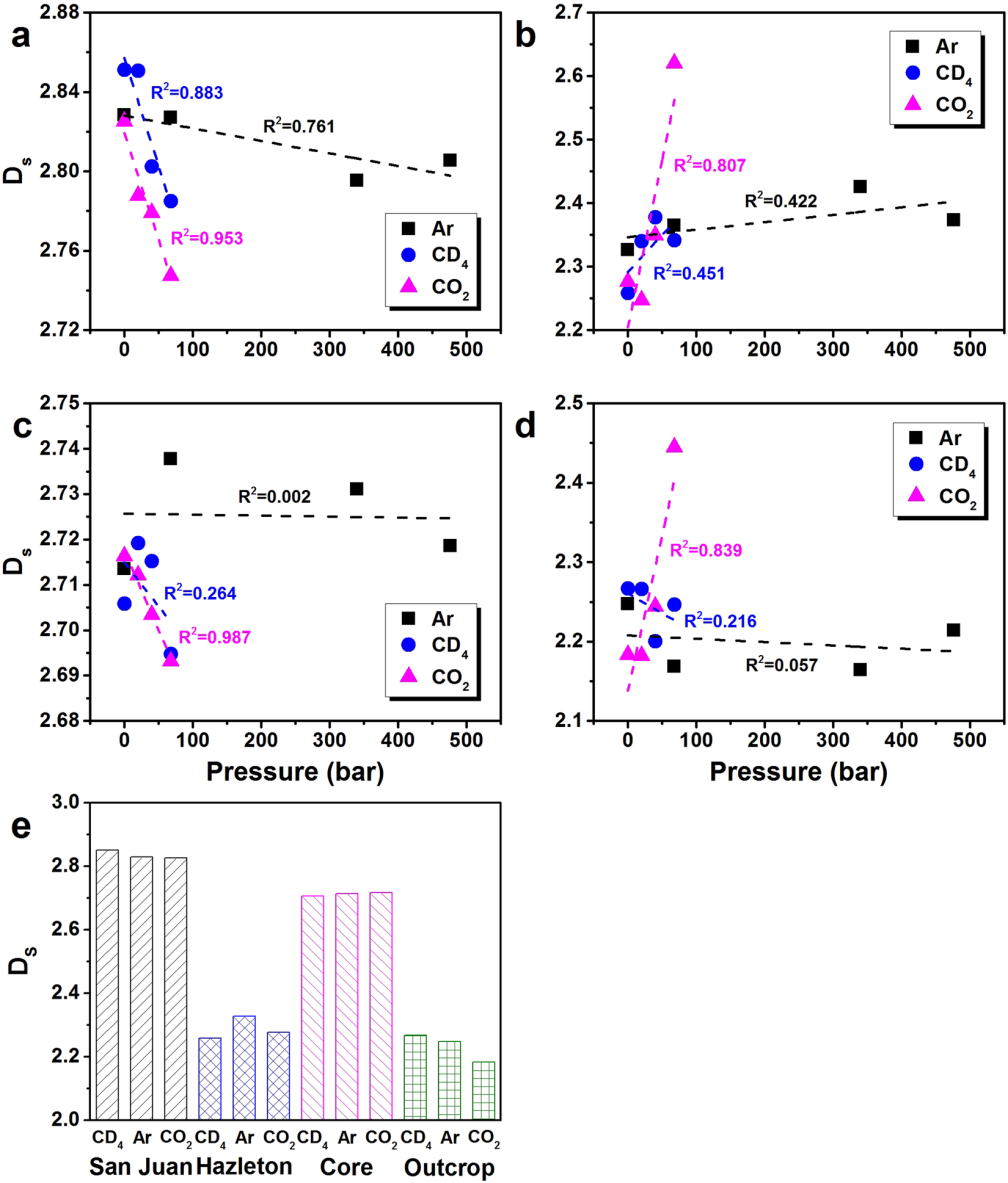
Evolution of fractal dimension based on argon, methane and CO_2_ penetrations. (**a**) San Juan coal; (**b**) Hazleton coal; (**c**) Marcellus drilled core shale; (**d**) Marcellus outcrop shale; (**e**) vacuum conditions for four samples.

The solid straight lines in Fig. 1 are the modeled power law scattering results, where the slope of these lines represents the fractal feature of tested samples^59^. Figure 4 shows the estimated surface fractal dimension *D*_s_ as function of pressure using argon, methane and CO_2_ for the tested four rock samples. The value of *D*_s_ follows the order of San Juan coal > Marcellus drilled core shale > Hazleton anthracite > Marcellus outcrop shale. These results indicate that the San Juan coal and Marcellus drilled core shale have more complex pore structures than other two samples. The values of estimated *D*_s_ have negligible correlation with pressure for argon injection at pressure up to 500 bar for all four samples except San Juan coal as illustrated as black squares in Fig. 4. It suggests that no obvious pore micro-damages were observed with hydrostatic argon injection. This means the localized pore surface morphology has no obvious correlation with argon pressurization.

For methane adsorption, only San Juan coal shows a decrease of *D*_s_ with increasing methane pressure (Fig. 4a) and no apparent correlation between methane pressure and estimated surface fractal dimensions were observed for other three rocks with pressure range from 0 to 68 bar (Fig. 4b–d). However, the scattering intensity *I*(*Q*) continuously decreased with increasing methane pressure for all four samples (Figs 1c, S1 c, S2 c and S3c). These results suggest that the progressive methane adsorption on San Juan coal tends to continuously smooth the surface of the internal pores since the fractal dimension continuously decreases with elevated methane pressure. Based on the results of our previous study, the methane adsorption capacity increased with increasing methane pressure for San Juan coal^44^, where the methane sorptive sites continuous decreased. The decrease of methane sorptive sites could be caused by the decrease of *D*_s_ with increasing methane pressure as a result of increasing sorption capacity. In this situation, the specific surface area for methane adsorption decreased with increasing methane pressure. The methane molecule tends to be adsorbed on the relative rough surface, which has high *D*_s_ and could be micropores, with high sorptive energy at low equilibrium pressure for San Juan coal. However, the other three rock samples do not have the same phenomenon where the surface fractal dimension randomly varies with methane pressure. Based on the fractal results, it is hard to tell whether methane adsorption occurred in small or large pores, and whether the adsorption is a layering process or pore-filling process in these porous rocks. Further studies will be needed to evaluate the evolution of pore size distribution and estimation of average gas density in pores. These advanced microscale structural evolution knowledge will potentially shed the light on methane adsorption mechanism for porous rocks.

For CO_2_ injection, *D*_s_ decreased with increasing CO_2_ pressure for San Juan coal and Marcellus drilled core shale (Fig. 4a and c). On the other hand, *D*_s_ increased with increasing pressure for Hazleton coal and Marcellus outcrop shale (Fig. 4b and d). Similar to methane injection, the scattering intensity also shows a continuously decrease with increasing CO_2_ pressure at this pressure range for all four samples (Figs 1d, S1 d, S2 d and S3d).

From Fig. 4, both methane and CO_2_ injections induced a decrease of surface fractal dimension value for San Juan coal which suggests that the internal surface of pores were smoothed by gas adsorption. Except San Juan coal, no obvious relationship was observed for the effects of methane and CO_2_ adsorption on the surface fractal for the other three rock samples. This may be due to the complex microstructure of the natural rocks. The complex structure of rocks will directly influence the adsorption mechanisms, either layering or pore-filling adsorptions. Certainly, more details on the gas adsorption mechanism on fractal rocks will be required to refine the understanding of the surface fractal changes with gas adsorption. Although, no definite relationship was found between the adsorption and surface fractal behavior, but it was confirmed that the adsorption will change the surface morphology as compared to argon injection.

A significant increase of *D*_s_ was observed when CO_2_ pressure is up to 68 bar for Hazleton coal and Marcellus outcrop shale (Fig. 4b and d). It is noted that CO_2_ is liquefied at 68 bar under room temperature based on CO_2_ phase diagram. It suggests that the CO_2_ adsorption mechanism is fundamentally different between gas and liquid phases, which has a huge effect on the pore surface with relative higher TOC for tested coal and shale, respectively. The liquid CO_2_ was highly densified in accessible pores where the scattering intensity has an obvious decrease with the phase change from gas to liquid for CO_2_ (Figs 1d, S1 d, S2 d and S3d). Compared to inaccessible pores, the pores with low heterogeneity or irregularity tend to be interconnected with each other which are accessible to the penetrating fluids. These accessible pores with relative smooth surface (relative small *D*_s_) becomes “invisible” from neutron beam during liquid CO_2_ densification due to the SLD of liquid CO_2_ approached the SLD of solid matrix. Although *D*_s_ significant increased for Hazleton coal and Marcellus outcrop shale under CO_2_ phase change, the surface fractal dimension of inaccessible pores is still higher than *D*_s_ of CO_2_ penetration at 68 bar (Fig. 4b and d). It indicates that a small portion of accessible pores was not filled by liquid CO_2_, where the ZAC condition was not reached at 68 bar for CO_2_ for these two samples.

Based on the sequential of SANS experiments, scattering intensity at vacuum condition was detected initially for all these samples. And there are two vacuum tests after argon and before CO_2_ penetrations, respectively. The surface fractal dimension *D*_s_ shows a slightly variation for San Juan and Hazleton coals, while there is negligible change of *D*_s_ for Marcellus drilled core shale (Fig. 4e). It is interesting that *D*_s_ decreased for Marcellus outcrop shale after both pressure and sorption effects. These findings suggest the change of fractal dimension before and after both pressurization and methane adsorption could be sample dependent.

## Summary

SAXS, SANS and low-pressure N_2_ sorption experiments were conducted to investigate the fractal characteristics for total, accessible and inaccessible pores for two coals and two shales. Fractal dimension was estimated by the combination modeling of power law and polydisperse sphere pore (PDSP) scatterings for SAXS and SANS, while the fractal Frenkel-Halsey-Hill (FHH) model was used to estimate the fractal dimension using low-pressure N_2_ sorption data. Uniquely, the evolution of fractal dimension was probed and quantified under *in situ* hydrostatic pressurization and gas sorption environments. Based on previous mentioned fractal characterization, several conclusions can be drawn below:

1. *D*_s_ of inaccessible pores are greater than that for total pores based on SANS results for all tested samples (Fig. 3a–d). *D*_s_ of accessible pores for all linear sections estimated by low-pressure N_2_ adsorption experiment are smaller than SANS-estimated *D*_s_ of total pores for only San Juan coal (Fig. 3a). The *D*_s_ of accessible pores are unexpected greater than that for total pores for Marcellus outcrop shale (Fig. 3d).
2. *D*_s_ of accessible pores estimated by N_2_ desorption isotherm is greater than that for N_2_ adsorption isotherm for each linear section for each sample (Fig. 3a, c and d).
3. There are negligible consistent between pore fractal dimension *D*_p_ estimated by SAXS data (Fig. 3e) and the *D*_p_ estimated using low-pressure N_2_ sorption data at high relative pressure (Fig. 3a, c and d).
4. *D*_s_ shows a slightly negative correlation with argon injecting pressure for only San Juan coal among tested four samples (Fig. 4a), which indicates that pressurization effect for fractal characteristics may be sample dependent.
5. *D*_s_ decreased with increasing of methane and CO_2_ injecting pressure for San Juan coal and Marcellus drilled core shale, which both have relative high *D*_s_ values (Fig. 4a and c). While *D*_s_ significantly increased when CO_2_ become liquid for Hazleton coal and Marcellus outcrop shale, which both have relative low *D*_s_ values (Fig. 4b and d).
6. *D*_s_ shows very small variation after methane and argon penetrations for all tested samples except Marcellus outcrop shale, where *D*_s_ slightly decreased after pressurization and sorption effects (Fig. 4e).

## Methods

### Materials

Two coal samples were collected from two underground coal mines: one sub-bituminous coal is from the northern San Juan basin in New Mexico and the other is an anthracite sample from Hazleton in Pennsylvania. One shale sample was obtained from a drilled well of Marcellus shale reservoir in Pennsylvania, while another one was collected from an outcrop mine of Marcellus shale in Pennsylvania. All rocks were crushed to powders with particle size of ~0.5 mm and put into oven for drying 24 h before characterizing experiments. The X-ray diffraction (XRD) results for these samples are shown in Table S1. San Juan and Hazleton coals have higher TOC compared to shale samples, where the TOC is 70.73% for San Juan coal and 91.14% for Hazleton coal. Marcellus drilled core shale has the lowest TOC (2.72%) and there is 9.52% organic matter for Marcellus outcrop shale. The XRD compositions were used to estimate the effective SLD for both SAXS and SANS characterizations.

### Small angle X-ray scattering

The SAXS experiment was conducted using a PANalytical Empyrean θ-θ diffractometer in Materials Research Institute (MRI) at The Pennsylvania State University. Powder samples were used with particle size of ~0.5 mm to detect scattering intensity as function of scattering vector at room temperature and vacuum condition. The X-ray beam was generated by Cu Kα radiation with wavelength of 1.54 Å and the beam went through a divergence slit and detected by a PIXcel3D detector in 1D scanning mode. The effective range of scattering vector varies between 0.00425 and 0.35555 Å^−1^. This wide range of scattering vector covers a relatively wide spectrum of pore size including partial macropores, whole mesopores and partial micropores. In order to estimate the true sample scattering intensity, the raw scattering data were processed by subtracting the background scattering intensity using the empty sample holder. The processed scattering data were used in subsequent data analyses.

### Small angle neutron scattering

The SANS experiment was conducted using the general-purpose small-angle neutron scattering diffractometer (GP-SANS) in High Flux Isotope Reactor (HFIR) at Oak Ridge National Laboratory (ORNL)^60^. Two coal and two shale samples with particle size of ~0.5 mm were tested under a vacuum to quantify the background scattering intensity. For each rock sample, the deuterated methane (CD_4_) was injected at 20 bar, 40 bar, 68 bar and zero average contrast (ZAC) pressure (340 bar for San Juan coal and 476 bar for Hazleton coal, Marcellus drilled core and outcrop shales) for each sample to monitor scattering intensity changes^43^. After the completion of the CD_4_ cycle, argon was injected at 68 bar, 340 bar and 476 bar respectively to check the pore stability under gas pressurization. For argon injection, the scattering intensities were measured and recorded at each pressure step. After the argon cycle, CO_2_ was injected at 20 bar, 40 bar and 68 bar for each rock sample to monitor scattering intensity changes. Each sample was vacuumed before the next gas injection sequence. The neutron wavelength was set at 6 Å and the wavelength spread was set at 0.13, as well as the distances of 2D detector were chosen at 0.3 and 18.5 m which cover an overall range of scattering vectors (0.00305 < *Q* < 0.5 Å^−1^) in the partial macorpore, whole mesopore and partial micropore regions. In addition, all the detected scattering intensities were normalized to absolute scattering intensities by using the effective thickness of powder samples and the secondary standard^61^. A schematic for both SAXS and SANS is shown in Fig. S6.

### Low-pressure N_2_ adsorption

The low-pressure N_2_ adsorption experiment was conducted using a ASAP 2020 Plus Physisorption technique in MRI at The Pennsylvania State University. Powder samples were measured by low-pressure N_2_ adsorption-desorption at −196 °C.

### Estimation of fractal dimension using SAXS/SANS

Both coal and shale are heterogeneous and anisotropic fractal system. The fractal feature of rock-pore structure can be evaluated and quantitatively described by different values of fractal dimension^62^. In order to estimate fractal dimension for neutron and X-ray scatterings, the scattering intensities were modeled as:

1 $$I(Q)={C}_{{\rm{p}}}{Q}^{-\alpha }+N{({\rho }_{{\rm{s}}}^{\ast }-{\rho }_{{\rm{p}}}^{\ast })}^{2}\int {V}^{2}(r)D(r)P(Q,r)dr+{I}_{{\rm{B}}}$$

where the first term *C*_p_ *Q* ^−*α*^, called the power law scattering^59^, is the scattering intensity depending on the pore morphology due to the pore surface roughness; *C*_p_ is the contrast factor which is *Q*-independent and depends on both the specific surface area of pore-matrix interface and the scattering contrast between pore and solid matrix; *α* is the power law exponent which describes the fractal nature of porous system; the second term is the polydisperse sphere pore (PDSP) scattering representing the polydispersity of pores in a porous system^63^; *N* is the pore number density; ${\rho }_{{\rm{s}}}^{\ast }$ is the scattering length density (SLD) of solid matrix; $\,{\rho }_{{\rm{p}}}^{\ast }$ is the SLD of pore; *r* is the sphere radius; *V*(*r*) is the spherical volume; *D*(*r*) is the pore size distribution; *P*(*Q*, *r*) is the spherical form factor; *I*_B_ is the background. In the low *Q* region for both SAXS and SANS, scattering intensity *I*(*Q*) and scattering vector *Q* has a power law correlation which was used to estimate the fractal dimension of the tested samples in this study.

Based on the fractal theories, there are two types of fractal characteristics: (1) The surface area of pores (the pore-matrix boundary) has power correlation with the length scale of building blocks where the surface fractal dimension *D*_s_ varies from 2 to 3; (2) The mass (volume) of grains has power correlation with the length scale of building blocks where the mass fractal dimension *D*_m_ is smaller than 3^64^. The *D*_m_ may be inversely interpreted as the volume of pores having power correlation with the length scale of building blocks. In this situation, the mass fractal dimension *D*_m_ is the same as so-called pore fractal dimension *D*_p_ for porous rocks. For both SAXS and SANS experiments, when power law exponent *α* ranges between 3 and 4, the scattering profile shows surface fractal where *D*_s_ = 6 − *α* ^59^. For a mass fractal or pore fractal, the value of *α* is smaller than 3 where *D*_m_ = *α* or *D*_p_ = *α* ^47^. It is important to note that both coal and shale rocks have either surface fractal or mass fractal or combination of these two at a certain pore size range.

### Estimation of fractal dimension using low-pressure N_2_ adsorption

Many methodologies have been developed to estimate the fractal dimension based on adsorption techniques such as fractal Brunauer-Emmett-Teller (BET) model^65^, thermodynamic method^66^ and fractal Frenkel-Halsey-Hill (FHH) model^58^. The FHH model is the most applicable method to estimate fractal dimension due to the estimation only needs one single gas adsorption isotherm. The fractal FHH equation can be expressed as^51^:

2 $$V=A{[\mathrm{ln}(\frac{{P}_{0}}{P})]}^{s}+B$$

where *V* is the total sorption capacity; *A* and *B* are characteristic constants; *P* is the equilibrium pressure; *P*_0_ is the saturated vapor pressure; *s* is an exponent which represents the fractal nature of object. There are two different sorption mechanisms for multilayer N_2_ adsorption^57^. When the adsorption occurred from monolayer to just several multilayers, the N_2_ sorption is dominated by the Van der Waals force between gas and pore surface where the fractal exponent *s* and the surface fractal dimension *D*_s_ has a relationship as *s* = (*D*_s_ − 3)/3. However, the effect of Van der Waals force reduced and the capillary condensation (surface tension between liquid and gas) becomes prevalent when pore-filling adsorption becomes the dominating mechanism where the relationship between *s* and *D*_s_ changes to *s* = *D*_s_ − 3^57^. Based on these two relationships, *D*_s_ can be easily estimated by the linear fitting of the correlation between ln*V* and ln[ln(*P*_0_/*P*)]. Since these two sorption mechanisms may simultaneous exist not only in different size of pores but also in different relative pressure range for most of adsorption experiments^67^, it is crucial to select the appropriate equation of fractal dimension estimation either *s* = (*D*_s_ − 3)/3 or *s* = *D*_s_ − 3 for a specific relative pressure range to get an accurate and reasonable value of *D*_s_. It was found that there are two to three obvious linear sections from low to high relative pressure on ln*V*-ln[ln(*P*_0_/*P*)] plot for both adsorption and desorption isotherms (Figs 3b, S4 b and S5b), which are usually shown in previous N_2_ sorption isotherms for both coal and shale^6, 13, 31, 67, 68^. The regression of all linear regions showed good fit where R^2^ are greater than 0.998 for all tested sample. *D*_s_ estimated by the relationship *s* = (*D*_s_ − 3)/3 on the linear section at lower relative pressure are unreasonably smaller than 2 for all these samples. It indicates that capillary condensation effect may be dominant at both low and high relative pressure for these coal and shale samples, where the relationship *s* = *D*_s_ − 3 should be used to estimate fractal dimension for all the linear regions over the entire pressure range in this study.

## Electronic supplementary material

Supplementary Information
