# Supplementary material for: Fractal evolution under in situ pressure and sorption conditions for coal and shale
Source: Sci Rep. 2017 Aug 21;7:8971. doi: 10.1038/s41598-017-09324-9 (PMC5566402; doi:10.1038/s41598-017-09324-9)
Supplement: Supplementary file 1 — Supplementary Information [file 41598_2017_9324_MOESM1_ESM.pdf]

# Fractal evolution under *in situ* pressure and sorption conditions for coal and shale

Rui Zhang[1], Shimin Liu[1,*], Yang Wang[2,3]

[1]Department of Energy and Mineral Engineering, G[3] Center and Energy Institute, The Pennsylvania State University, University Park, PA 16802, USA
[2]Key Laboratory of Coalbed Methane Resources and Reservoir Formation Process, Ministry of Education, Xuzhou, Jiangsu 221008, China
[3]School of Resources and Earth Science, China University of Mining and Technology, Xuzhou, Jiangsu 221116, China

Corresponding Author
[*]Tel. No.: +1 8148634491; Fax No.: +1 8148653248; Email: szl3@psu.edu

## Supplementary

### Figures

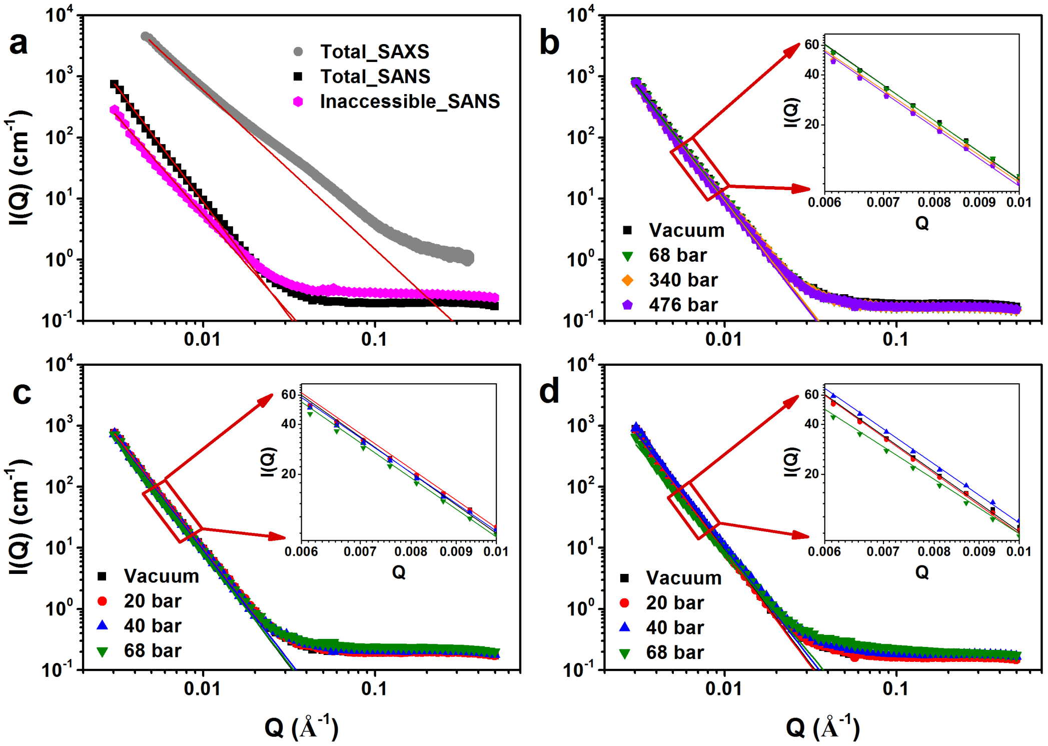


**Figure S1. Scattering intensities of Hazleton coal.** (**a**), scattering intensities of total pores detected by SAXS and SANS, scattering intensities of inaccessible pores detected by SANS; (**b**),

scattering intensities during Ar penetration; (**c**), scattering intensities during $CD_4$ penetration; (**d**), scattering intensities during $CO_2$ penetration. (Note: The solid lines are modeled power-law scattering intensities for fractal dimension determination.)

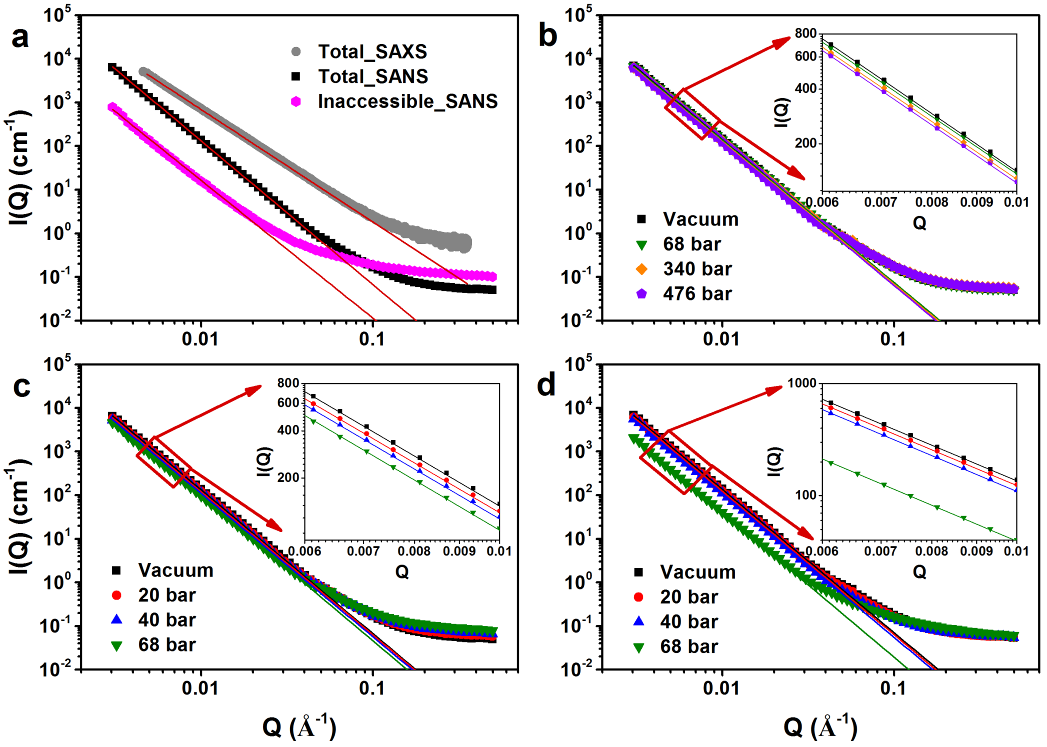


**Figure S2. Scattering intensities of Marcellus drilled core shale.** (**a**), scattering intensities of total pores detected by SAXS and SANS, scattering intensities of inaccessible pores detected by SANS; (**b**), scattering intensities during Ar penetration; (**c**), scattering intensities during $CD_4$ penetration; (**d**), scattering intensities during $CO_2$ penetration. (Note: The solid lines are modeled power-law scattering intensities for fractal dimension determination.)

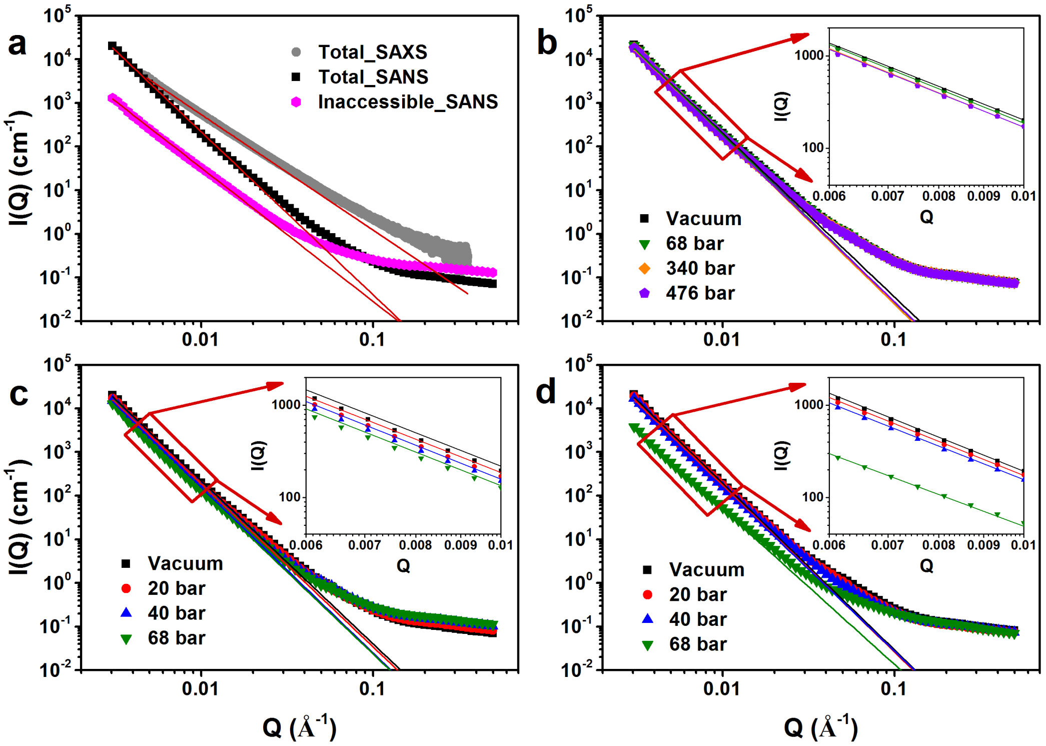


**Figure S3. Scattering intensities of Marcellus outcrop shale.** (**a**), scattering intensities of total pores detected by SAXS and SANS, scattering intensities of inaccessible pores detected by SANS; (**b**), scattering intensities during Ar penetration; (**c**), scattering intensities during $CD_4$ penetration; (**d**), scattering intensities during $CO_2$ penetration. (Note: The solid lines are modeled power-law scattering intensities for fractal dimension determination.)

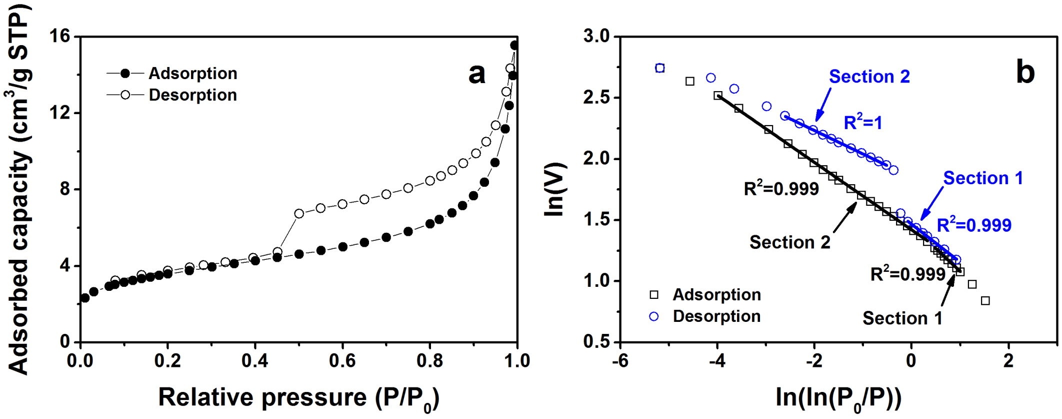


**Figure S4. Low-pressure $N_2$ sorption of Marcellus drilled core shale.** (**a**), low-pressure $N_2$ adsorption-desorption isotherms; (**b**), $\ln(V)$ - $\ln(\ln(P_0/P))$ plot of $N_2$ isotherms for fractal determination.

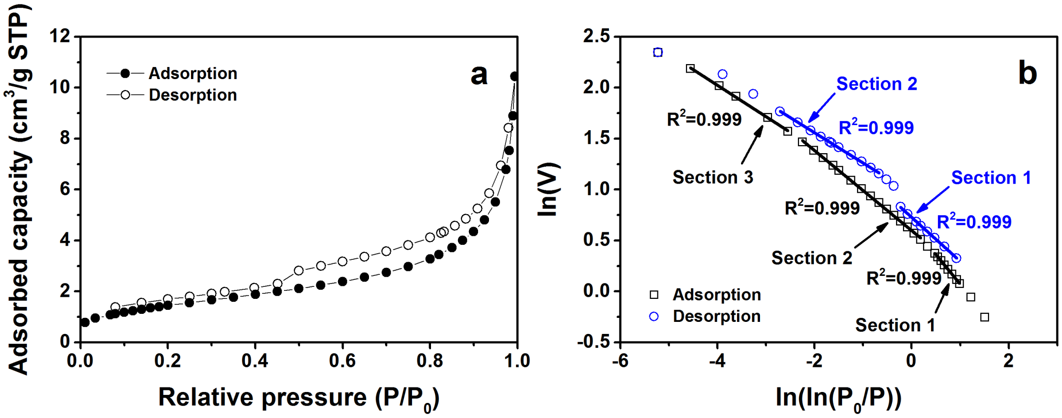


**Figure S5. Low-pressure $N_2$ sorption of Marcellus outcrop shale.** (**a**), low-pressure $N_2$ adsorption-desorption isotherms; (**b**), $\ln(V)$ - $\ln(\ln(P_0/P))$ plot of $N_2$ isotherms for fractal determination.

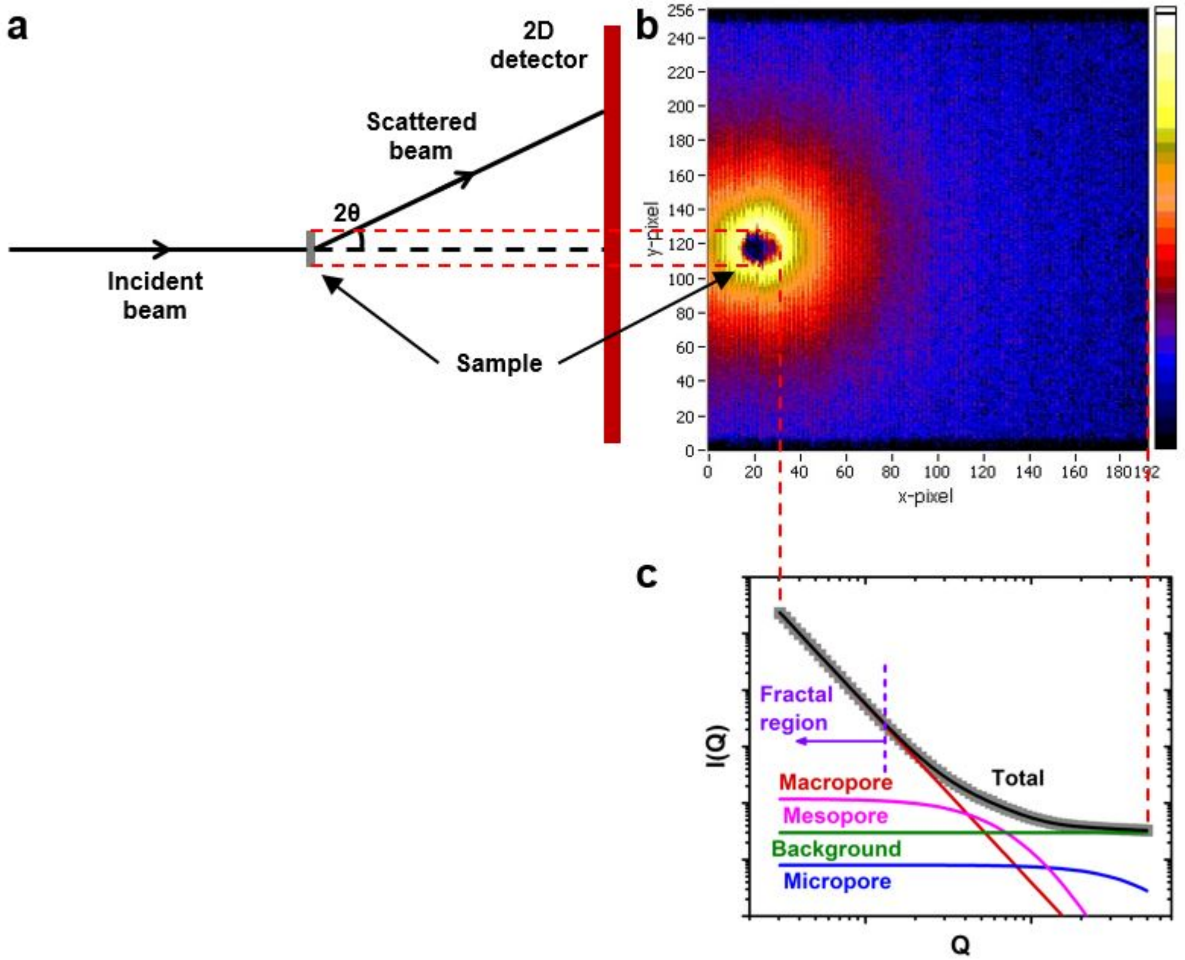


**Figure S6. A schematic of SANS (SAXS) data reduction process.** (**a**), a simplified schematic of SANS (SAXS) instrument where the incident neutron (electron) beam is generated by neutron fission reactor or spallation source (Cu Kα radiation) and transmits through the sample and is elastically scattered (scattered and absorption) $2\theta$ scattering angle; (**b**), a fully counted 2D image obtained from the 2D detector; (**c**), a statistically reduced 1D log-log plot with modeling illustration.

## Tables

**Table S1. XRD results for four tested samples.**

| | | | | | | | | |
|---|---|---|---|---|---|---|---|---|
| San Juan coal | Quartz | Kaolinite | TOC | | | | | |
| | 4.71% | 24.56% | 70.73% | | | | | |
| Hazleton coal | Tobelite | TOC | | | | | | |
| | 8.86% | 91.14% | | | | | | |
| Marcellus drilled core shale | Quartz | Muscovite | Albite | Clinochlore | Dolomite | Pyrite | Calcite | TOC |
| | 27.63% | 31.03% | 17.41% | 12.16% | 5.16% | 3.02% | 0.87% | 2.72% |
| Marcellus outcrop shale | Quartz | Muscovite | TOC | | | | | |
| | 74.56% | 15.92% | 9.52% | | | | | |

**Table S2. Low-pressure $N_2$ sorption results of fractal dimension for four tested samples.**

| Sample | $N_2$ adsoprtion | | | | | | | | | $N_2$ desorption | | | | | |
|---|---|---|---|---|---|---|---|---|---|---|---|---|---|---|---|
| | Region 1 | | | Region 2 | | | Region 3 | | | Region 1 | | | Region 2 | | |
| | s | $D_s$ | $P/P_0$ range | s | $D_s$ | $P/P_0$ range | s | $D_s$ | $P/P_0$ range | s | $D_s$ | $P/P_0$ range | s | $D_s$ | $P/P_0$ range |
| San Juan coal | -0.54 | 2.46 | 0.067-0.200 | -0.46 | 2.54 | 0.249-0.850 | -0.30 | 2.70 | 0.874-0.981 | -0.44 | 2.56 | 0.080-0.396 | -0.31 | 2.69 | 0.700-0.946 |
| Marcellus drilled core shale | -0.37 | 2.63 | 0.066-0.200 | -0.28 | 2.72 | 0.249-0.982 | / | / | / | -0.31 | 2.69 | 0.080-0.396 | -0.19 | 2.81 | 0.550-0.929 |
| Marcellus outcrop shale | -0.58 | 2.42 | 0.068-0.200 | -0.39 | 2.61 | 0.300-0.900 | -0.31 | 2.69 | 0.925-0.990 | -0.43 | 2.57 | 0.080-0.451 | -0.30 | 2.70 | 0.600-0.936 |

**Table S3. SAXS results of fractal dimension for four tested samples.**

| Sample | $\alpha$ | $D_m$ ($D_p$) | $Q$ range (Å$^{-1}$) |
|---|---|---|---|
| San Juan coal | 2.58 | 2.58 | 0.005-0.36 |
| Hazleton coal | 2.60 | 2.60 | 0.005-0.36 |
| Marcellus drilled core shale | 2.58 | 2.58 | 0.005-0.36 |
| Marcellus outcrop shale | 2.65 | 2.65 | 0.005-0.36 |

**Table S4. SANS results of fractal dimension by argon injection for four tested samples.**

| Sample | Vacuum | | 68 bar | | 340 bar | | 476 bar | | $Q$ range (Å$^{-1}$) |
|---|---|---|---|---|---|---|---|---|---|
| | $\alpha$ | $D_s$ | $\alpha$ | $D_s$ | $\alpha$ | $D_s$ | $\alpha$ | $D_s$ | |
| San Juan coal | 3.17 | 2.83 | 3.17 | 2.83 | 3.20 | 2.80 | 3.19 | 2.81 | 0.003-0.50 |
| Hazleton coal | 3.67 | 2.33 | 3.64 | 2.36 | 3.57 | 2.43 | 3.63 | 2.37 | 0.003-0.50 |
| Marcellus drilled core shale | 3.29 | 2.71 | 3.26 | 2.74 | 3.27 | 2.73 | 3.28 | 2.72 | 0.003-0.50 |
| Marcellus outcrop shale | 3.75 | 2.25 | 3.83 | 2.17 | 3.84 | 2.16 | 3.79 | 2.21 | 0.003-0.50 |

**Table S5. SANS results of fractal dimension by methane injection for four tested samples.**

| Sample | Vacuum | | 20 bar | | 40 bar | | 68 bar | | ZAC | | $Q$ range (Å$^{-1}$) |
|---|---|---|---|---|---|---|---|---|---|---|---|
| | $\alpha$ | $D_s$ | $\alpha$ | $D_s$ | $\alpha$ | $D_s$ | $\alpha$ | $D_s$ | $\alpha$ | $D_s$ | |
| San Juan coal | 3.15 | 2.85 | 3.15 | 2.85 | 3.20 | 2.80 | 3.22 | 2.78 | 3.00 | 3.00 | 0.003-0.50 |

| | | | | | | | | | | | |
|---|---|---|---|---|---|---|---|---|---|---|---|
| Hazleton coal | 3.74 | 2.26 | 3.66 | 2.34 | 3.62 | 2.38 | 3.66 | 2.34 | 3.21 | 2.79 | 0.003-0.50 |
| Marcellus drilled core shale | 3.29 | 2.71 | 3.28 | 2.72 | 3.28 | 2.72 | 3.31 | 2.69 | 3.16 | 2.84 | 0.003-0.50 |
| Marcellus outcrop shale | 3.73 | 2.27 | 3.73 | 2.27 | 3.80 | 2.20 | 3.75 | 2.25 | 3.06 | 2.94 | 0.003-0.50 |

**Table S6. SANS results of fractal dimension by $CO_2$ injection for four tested samples.**

| Sample | Vacuum | | 20 bar | | 40 bar | | 68 bar | | $Q$ range (Å$^{-1}$) |
|---|---|---|---|---|---|---|---|---|---|
| | $\alpha$ | $D_s$ | $\alpha$ | $D_s$ | $\alpha$ | $D_s$ | $\alpha$ | $D_s$ | |
| San Juan coal | 3.17 | 2.83 | 3.21 | 2.79 | 3.22 | 2.78 | 3.25 | 2.75 | 0.003-0.50 |
| Hazleton coal | 3.72 | 2.28 | 3.75 | 2.25 | 3.65 | 2.35 | 3.38 | 2.62 | 0.003-0.50 |
| Marcellus drilled core shale | 3.28 | 2.72 | 3.29 | 2.71 | 3.30 | 2.70 | 3.31 | 2.69 | 0.003-0.50 |
| Marcellus outcrop shale | 3.82 | 2.18 | 3.82 | 2.18 | 3.76 | 2.24 | 3.56 | 2.44 | 0.003-0.50 |
